# Supplementary material for: TFIIE orchestrates the recruitment of the TFIIH kinase module at promoter before release during transcription
Source: Nat Commun. 2019 May 7;10:2084. doi: 10.1038/s41467-019-10131-1 (PMC6504876; doi:10.1038/s41467-019-10131-1)
Supplement: Supplementary file 1 — Supplementary Information [file 41467_2019_10131_MOESM1_ESM.pdf]

Supplementary Informations

TFIIE orchestrates the recruitment of the TFIIH kinase module at promoter before release during transcription.

by Compe et al.

This file includes Supplementary Table 1 and Supplementary Figs. 1 to 7

Supplementary Table 1: Key Resources Table

REAGENTS and RESOURCES	SOURCE	IDENTIFIER
**Antibodies**		
goat anti-mouse kappa-HRP	Southern Biotech	1050-05
mouse monoclonal anti β-actin	IGBMC Antibody Facility	clone 2D7
mouse monoclonal anti CDK7	IGBMC Antibody Facility	clone 2F8
mouse monoclonal anti Cyclin H	IGBMC Antibody Facility	clone 2D4
mouse monoclonal anti-Flag tag	Sigma-Aldrich	F1804 ; RRID: AB_262044
rabbit polyclonal anti-Flag tag	Sigma-Aldrich	F7425; RRID: AB_439687
mouse monoclonal anti-rabbit light chain-HRP	Jackson Immunoresearch	211-032-171
rabbit polyclonal anti p33 (TFIIB) (C-18)	Santa Cruz	sc-225, RRID: AB_2114380
mouse monoclonal anti p52	IGBMC Antibody Facility	clone 1D11
mouse monoclonal anti p62 (immunoblotting)	IGBMC Antibody Facility	clone 3C9
rabbit polyclonal anti p62 (immunoprecipitation)	Santa Cruz	sc-292; RRID: AB_2114675
mouse monoclonal anti RAP30	IGBMC Antibody Facility	clone 1E4
mouse monoclonal anti RAP74	IGBMC Antibody Facility	clone 2A3
rabbit polyclonal anti RARα	IGBMC Antibody Facility	clone 115
RARα phospho serine77 antibody	IGBMC Antibody Facility	clone 27α1G3
RNA pol II CTD phospho ser5 antibody	Active Motif	clone 3E8; RRID: AB_10615822
RNA pol II CTD phospho ser2 antibody	Active Motif	Clone 3E10; RRID: AB_2687450
mouse monoclonal anti RPB1	IGBMC Antibody Facility	clone 7C2
rabbit polyclonal anti RPB2	Abcam LTD	ab10338 ; RRID: AB_2167495
rabbit polyclonal anti SPT5 (H-300)	Santa Cruz	sc-28678; RRID: AB_668824
rabbit polyclonal anti TAF1	Millipore	ABE42; RRID: AB_10863146
rabbit polyclonal anti TAF4	Active motif	ab63910 ; RRID: AB_1143271
mouse monoclonal anti TBP	IGBMC Antibody Facility	clone 3G3
mouse monoclonal anti TFIIEα	IGBMC Antibody Facility	clone 2A1
mouse monoclonal anti TFIIEβ	IGBMC Antibody Facility	clone 1C2
rabbit polyclonal anti XPB (immunoprecipitation)	Santa Cruz	sc-293; RRID: AB_2262177
mouse monoclonal anti XPB (immunoblotting)	IGBMC Antibody Facility	clone 1B3
rabbit monoclonal anti XPD	Cell Signaling	(D3Z61) 11963
**Chemicals**		
ATP	GE Healthcare Europe	27-2056-61
ATPγS	Abcam LTD	AB138911
[γ-32P] ATP	Hartmann Analytic	SRP-501

CpA dinucleotide	TEBU-BIO SAS	O-31005-25
CTP	Life Technologies SAS	R0451
THZ1	Clinisciences	B4736
Triptolide	BIO-TECHNE- R&D SYSTEMS EUROPE	3253-1
UTP	GE Healthcare Europe	27-2086-61
Commercial Assays		
Anti-Flag M2 Affinity Gel	Sigma-Aldrich	Cat#A2220
Dynabeads M-280 Streptavidin	Invitrogen	Cat#11206D
Dynabeads Protein G	Invitrogen	Cat#10004D
LightCycler 480 SYBR Green Master Mix	Roche	Cat#04887352001
Paraformaldehyde 16%	Thermo Fisher	Cat#50-980-487
QuantiTect SYBR Green PCR kit	Qiagen	Cat#1037795
SuperScript II RT	Thermo Fisher	Cat#18064014
TALON metal affinity resin	Clonetech	Cat#635501
Cell Lines		
382BE (normal fibroblasts isolated from the mother of TTD379BE)	IGBMC Cell Culture Facility	ref. 1
TTD379BE (bearing the TFIIEβ/A150P point mutation)	IGBMC Cell Culture Facility	ref. 1
C3PV (control cell line for TTD28PV)	IGBMC Cell Culture Facility	ref. 2
TTD28PV (bearing the TFIIEβ/D187Y point mutation)	IGBMC Cell Culture Facility	ref. 1
TTD8PV father (control cell line for TTD8PV)	IGBMC Cell Culture Facility	ref. 3
TTD8PV (bearing the XPD/R112H point mutation)	IGBMC Cell Culture Facility	ref. 3
TTD12PV father (control cell line for TTD12PV)	IGBMC Cell Culture Facility	ref. 4
TTD12PV (bearing the XPD/R722W point mutation)	IGBMC Cell Culture Facility	ref. 4
U2OS IIEβ/WT (human osteosarcoma cell line)	ATCC	HTB-96
U2OS KI-IIEβ/A150P	this paper	N/A
Oligonucleotides		
gRNA IIEβ/A150P	GATAAGAAGGCCCTACTTAGG	
RT-PCR		
RARβ2	CCAGCAAGCCTCACATGTTTCCAA	TACACGCTCTGCACCTTTAGCACT
18S	TCAACTTTCGATGGTAGTCGCCGT	TCCTTGGATGTGGTAGCCGTTTCT
ChIP		
RARB2 proximal promoter	TGGTGATGTCAGACTAGTTGGGTC	GCTCACTTCCTACTACTTCTGTCAC
RARB2 exon 4	TCCAGCTGTCAGGAATGACAGGAA	TGAGATCGTCCAACTCAGCTGTCA
Recombinant DNA		
AdMLP DNA		ref. 5
pAK309		ref. 5

pET15b His-TFIIEα	this paper	N/A
pFlag-TFIIEβ/ WT, /A150P and /D187Y	this paper	N/A
pSK278-FlagXPD/WT, /R112H and /R722W		ref. 6
px2-Cas9WT(GFP)-Puro-gRNA IIEβ/A150P	this paper	N/A
**Software**		
ImageJ	NIH	https://imagej.nih.gov/ij/
**Other**		
Amersham Imager 600		GE Healthcare Life Sciences
Ligthcycler 480		Roche
Sonicator Q800R2		Qsonica
Thermomixer C		Eppendorf

References

1. Kuschal, C. et al. GTF2E2 Mutations Destabilize the General Transcription Factor Complex TFIIE in Individuals with DNA Repair-Proficient Trichothiodystrophy. *Am. J. Hum. Genet.* **98**, 627-42 (2016).
2. Botta, E. et al. Reduced level of the repair/transcription factor TFIIH in trichothiodystrophy. *Hum. Mol. Genet.* **11**, 2919-28 (2002).
3. Stefanini, M. et al. DNA repair investigations in nine Italian patients affected by trichothiodystrophy. *Mutat. Res.* **273**, 119-25. (1992).
4. Botta, E. et al. Analysis of mutations in the XPD gene in Italian patients with trichothiodystrophy: site of mutation correlates with repair deficiency, but gene dosage appears to determine clinical severity. *Am. J. Hum. Genet.* **63**, 1036-48 (1998).
5. Gerard, M. et al. Purification and interaction properties of the human RNA polymerase B(II) general transcription factor BTF2. *J. Biol. Chem* **266**, 20940-5. (1991).
6. Dubaele, S. et al. Basal transcription defect discriminates between xeroderma pigmentosum and trichothiodystrophy in XPD patients. *Mol. Cell* **11**, 1635-46 (2003).

Supplementary Figure 1

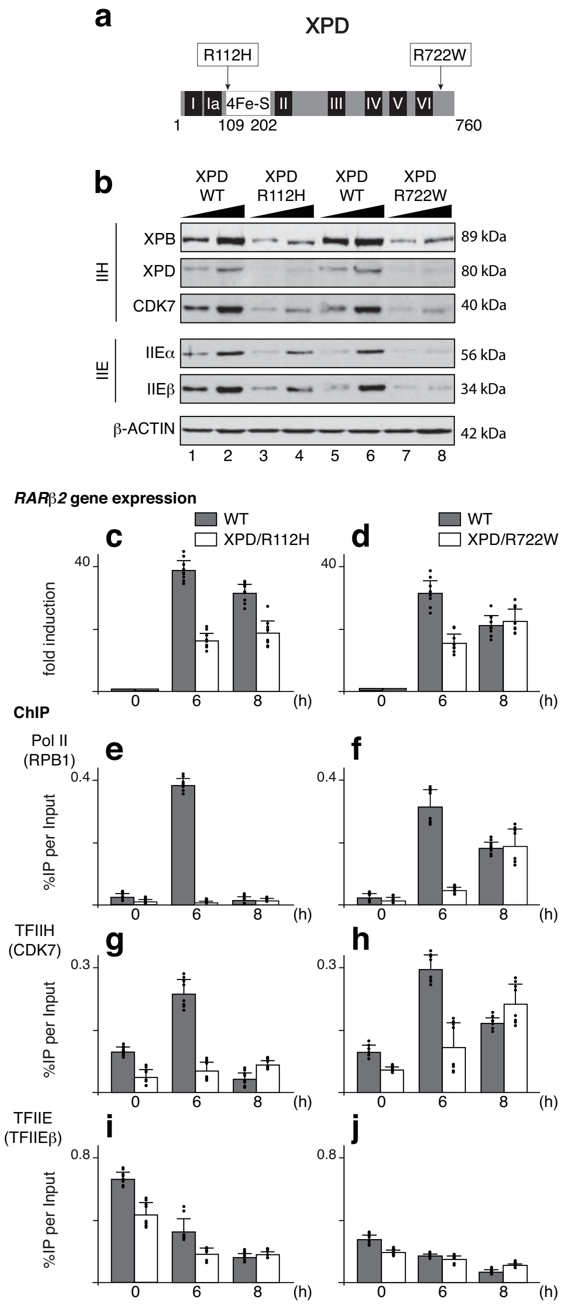

Supplementary Fig. 1

(**a**): Schematic representation of the human 760-aa XPD protein with the Fe-S iron sulfur-containing domain (4Fe-S, from 109 to 202) and the 7 (I-VI) helicase motifs. Amino acid changes (R112H and R722W) resulting from mutations commonly found in TTD patients are depicted.

(**b**): Anti-TFIIH (XPB, XPD and CDK7) and -TFIIE (IIEα and IIEβ) immunoblot analysis of increasing amounts of whole-cell lysates from fibroblasts of TTD patients with TFIIH-XPD/R112H, /R722W mutations and of unaffected parents. β-actin was used as loading control.

(**c-j**): Wild-type (grey boxes) and TTD fibroblasts (open boxes) with the mutation XPD/R112H (panels c, e, g and i) and /R722W (panels d, f, h and j) have been treated with t-RA (10µM). Relative RARβ2 gene expression have been measured after 0, 6 and 8h of t-RA treatment (panels c-d). The mRNA levels were normalized to the 18S RNA amount. The results (n=9, means±s.d.) are presented as n-fold induction relative to non-treated cells. ChIP experiments have been done 0, 6 and 8h post-t-RA treatment to analyse in normal and TTD fibroblasts the recruitment of Pol II (RPB1, panels e-f), TFIIH (CDK7, panels g-h) and TFIIE (TFIIEβ, panels i-j) at the *RARB2* proximal promoter. The values (n=8, means±s.d.) are expressed as percentage of immunoprecipitated DNA relative to the input. Source data are provided as a Source Data file.

Supplementary Figure 2

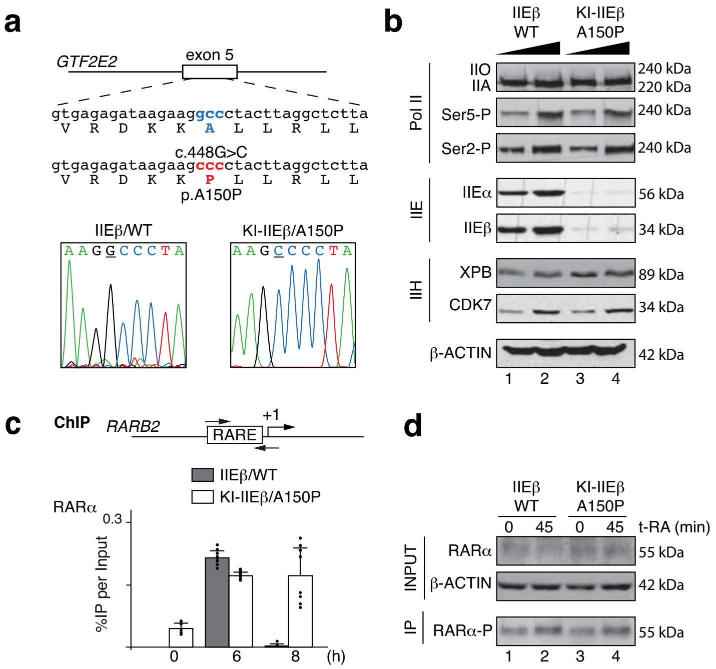

Supplementary Fig. 2

(**a**): Schematic representation of the *GTF2E2* gene encoding TFIIEβ and localization in exon 5 of the missense mutation (c.448G>C [p.Ala150Pro]) generated with *CRISPR/Cas9* methodology. Sequencing analysis confirmed full allelic targeting of the *GTF2E2* locus in a clone designed as KI-IIEβ/A150P; non-mutated cells (IIEβ/WT) have been used as control.

(**b**): Immunoblot analysis of Pol II (the hypo IIA- and hyper IIO- phosphorylated forms of RPB1, Ser2-P and Ser5-P), TFIIB (p33), TFIIE (IIEα and IIEβ), TFIIH (XPB and CDK7) has been done with increasing amounts of whole-cell extracts from IIEβ/WT and KI-IIEβ/A150P cells. β-actin was used as loading control.

(**c**): ChIP experiments have been done 0, 6 and 8h post-t-RA treatment to analyse in IIEβ/WT (grey boxes) and KI-IIEβ/A150P (open boxes) cells the recruitment of RARα to its response element (RARE, located in the proximal promoter). The values (n=8, means±s.d.) are expressed as percentage of immunoprecipitated (IP) DNA relative to the input.

(**d**): IIEβ/WT (grey boxes) and KI-IIEβ/A150P (open boxes) cells were treated 45min with t-RA (10µM). Whole cell extracts (2mg) were prepared and immunoprecipitated (IP) with purified mouse monoclonal antibodies recognizing RARα phosphorylated at Ser77. The eluates were then analysed by immunoblotting with antibodies against RARα. Phosphorylation blots are representative of two independent experiments. As a reference, immunoblot analysis with 50µg of whole cell lysates have been done with antibodies against RARα and β-actin. Source data are provided as a Source Data file.

Supplementary Figure 3

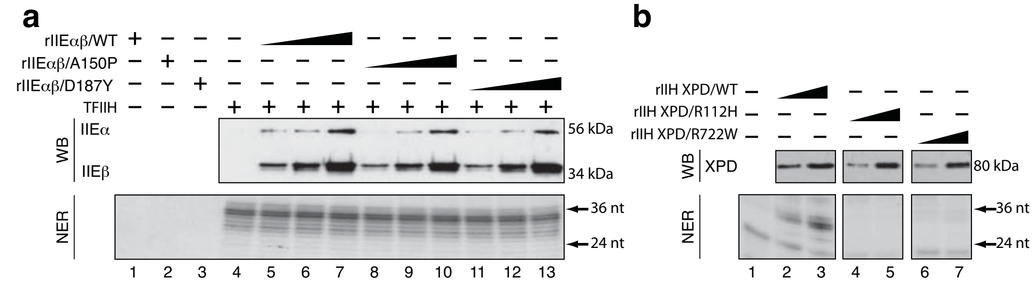

Supplementary Fig. 3

(**a**-**b**): Increasing amounts (as revealed by western blots) of rIIEs (**a**) and rIIHs (**b**) were added to an incision/excision (NER) assay using purified recombinant NER factors. The area of the gel containing the excision products is shown. The results are representative of three independent experiments. Source data are provided as a Source Data file.

Supplementary Figure 4

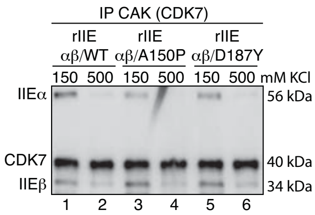

Supplementary Fig. 4

Recombinant CAK was immunoprecipitated (IP) via CDK7 and incubated with either rIIEβ/WT (lanes1-2), /A150P (lanes 3-4) or /D187Y (lanes 5-6) at 150mM and 500mM KCl. After washes, the co immunoprecipitated proteins were resolved by SDS-PAGE and blotted with antibodies against CDK7, TFIIEα and TFIIEβ. The results are representative of two independent experiments. Source data are provided as a Source Data file.

Supplementary Figure 5

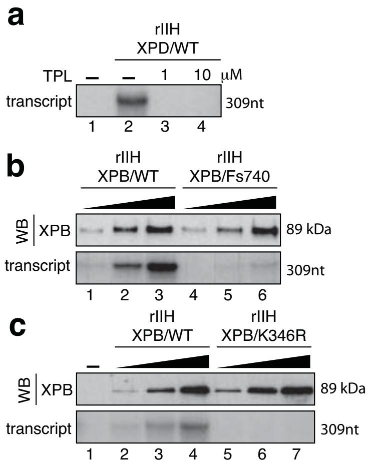

Supplementary Fig. 5

(**a**): *In vitro* reconstituted transcription system has been performed in presence of TPL (1 and 10µM). The transcription activity was stopped 45 min after addition of NTPs (including radiolabelled CTP). The length of the transcript (309nt) is indicated on the right side. The results are representative of two independent experiments.

(**b**): Increasing amounts (as revealed by Western Blots, WB) of rIIH XPB/WT (lanes 1-3) and /Fs740 (lanes 4-6) were added to an *in vitro* reconstituted transcription system. The transcription activity was stopped 45 min after addition of NTPs (including radiolabelled CTP). The length of the transcript (309nt) is indicated on the right side. The results are representative of two independent experiments.

(**c**): Increasing amounts (as revealed by Western Blots, WB) of rIIH XPB/WT (lanes 2-4) and /K346R (lanes 5-7) were added to an *in vitro* reconstituted transcription system, as described in panel B. The results are representative of two independent experiments. Source data are provided as a Source Data file.

Supplementary Figure 6

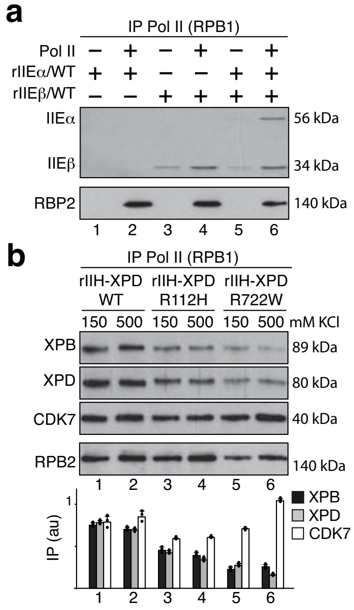

Supplementary Fig. 6

(**a**): Pol II interacts with TFIIE through its TFIIEβ subunit. Purified Pol II has been immunoprecipitated with antibodies raised against RPB1 and incubated (when indicated, +) with purified rIIEα/WT and/or rIIEβ/WT. After washes, immunoblot analysis has been done with antibodies against Pol II (RPB2), IIEα and IIEβ. The results are representative of two independent experiments.

(**b**): Interaction between Pol II and TFIIH recombinants. Immunoprecipitated Pol II (with antibody raised against RPB1) has been incubated with either rIIH-XPD/WT, /R112H or /R722W at 150 and 500mM KCl. After washes, the coimmunoprecipitated proteins were resolved by SDS-PAGE and blotted with antibodies against RPB2, XPB, XPD and CDK7. The immunoprecipitated signals (IP) for XPB, XPD and CDK7 were quantified and plotted in arbitrary units (au). The results are representative of two independent experiments. Source data are provided as a Source Data file.

Supplementary Figure 7

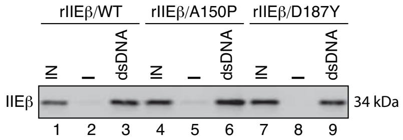

Supplementary Fig. 7

In vitro interaction between double-stand (ds) DNA and TFIIE. Each IIEβ recombinant (rIIEβ) was incubated with dsDNA cellulose. After extensive washes, the bound rIIEβ were detected by Western blotting. The results are representative of two independent experiments. Source data are provided as a Source Data file.
