# Supplementary material for: TFIIE orchestrates the recruitment of the TFIIH kinase module at promoter before release during transcription
Source: Nat Commun. 2019 May 7;10:2084. doi: 10.1038/s41467-019-10131-1 (PMC6504876; doi:10.1038/s41467-019-10131-1)
Supplement: Supplementary file 3 — Reporting Summary [file 41467_2019_10131_MOESM3_ESM.pdf]

nature research

Corresponding author(s): Emmanuel Compe
Jean-Marc Egly

Reporting Summary

Nature Research wishes to improve the reproducibility of the work that we publish. This form provides structure for consistency and transparency in reporting. For further information on Nature Research policies, see Authors & Referees and the Editorial Policy Checklist.

Statistical parameters

When statistical analyses are reported, confirm that the following items are present in the relevant location (e.g. figure legend, table legend, main text, or Methods section).

n/a	Confirmed	
☐	☒	The exact sample size (*n*) for each experimental group/condition, given as a discrete number and unit of measurement
☐	☒	An indication of whether measurements were taken from distinct samples or whether the same sample was measured repeatedly
☐	☒	The statistical test(s) used AND whether they are one- or two-sided<br>*Only common tests should be described solely by name; describe more complex techniques in the Methods section.*
☒	☐	A description of all covariates tested
☒	☐	A description of any assumptions or corrections, such as tests of normality and adjustment for multiple comparisons
☐	☒	A full description of the statistics including central tendency (e.g. means) or other basic estimates (e.g. regression coefficient) AND variation (e.g. standard deviation) or associated estimates of uncertainty (e.g. confidence intervals)
☒	☐	For null hypothesis testing, the test statistic (e.g. *F*, *t*, *r*) with confidence intervals, effect sizes, degrees of freedom and *P* value noted<br>*Give P values as exact values whenever suitable.*
☒	☐	For Bayesian analysis, information on the choice of priors and Markov chain Monte Carlo settings
☒	☐	For hierarchical and complex designs, identification of the appropriate level for tests and full reporting of outcomes
☒	☐	Estimates of effect sizes (e.g. Cohen's *d*, Pearson's *r*), indicating how they were calculated
☐	☒	Clearly defined error bars<br>*State explicitly what error bars represent (e.g. SD, SE, CI)*

Our web collection on statistics for biologists may be useful.

Software and code

Policy information about availability of computer code

Data collection	Image J (https://imagej.nih.gov/ij/) has been used to quantify signals obtained from in vitro experiments (Western Blots, run off assays, abortive transcription assays, kinase assays, PIC formation assays).
Data analysis	Excel (16.16.2, Microsoft) has been used to analyse the data.

For manuscripts utilizing custom algorithms or software that are central to the research but not yet described in published literature, software must be made available to editors/reviewers upon request. We strongly encourage code deposition in a community repository (e.g. GitHub). See the Nature Research guidelines for submitting code & software for further information.

Data

Policy information about availability of data

All manuscripts must include a data availability statement. This statement should provide the following information, where applicable:
- Accession codes, unique identifiers, or web links for publicly available datasets
- A list of figures that have associated raw data
- A description of any restrictions on data availability

All data generated during this study are included in this published article (and its suppementary information file).

Field-specific reporting

Please select the best fit for your research. If you are not sure, read the appropriate sections before making your selection.

☒ Life sciences ☐ Behavioural & social sciences ☐ Ecological, evolutionary & environmental sciences

For a reference copy of the document with all sections, see nature.com/authors/policies/ReportingSummary-flat.pdf

Life sciences study design

All studies must disclose on these points even when the disclosure is negative.

Sample size	Samples sizes were chosen on the basis of preliminary experiments. Typically, ChIp and RT-PCR studies result from three independent experiments performed in triplicates.
Data exclusions	No data were excluded.
Replication	ChIp and RT-PCR studies are representative of at least three independent experiments performed in triplicates as indicated by standard deviation (see Figure Legends). The in vitro experiments were repeated several times (as indicated in Figure legends) with similar results.
Randomization	No randomization was required for this study.
Blinding	Investigators were not blinded.

Reporting for specific materials, systems and methods

Materials & experimental systems

n/a	Involved in the study	
☒	☐	Unique biological materials
☐	☒	Antibodies
☐	☒	Eukaryotic cell lines
☒	☐	Palaeontology
☒	☐	Animals and other organisms
☒	☐	Human research participants

Methods

n/a	Involved in the study	
☒	☐	ChIP-seq
☒	☐	Flow cytometry
☒	☐	MRI-based neuroimaging

Antibodies

Antibodies used	Supplementary Table 1 provided with manuscript contains information on all antibodies used in the study.
Validation	See antibodies ID. Informations are available from http://antibodyregistry.org/

Eukaryotic cell lines

Policy information about cell lines

Cell line source(s)

The human primary fibroblasts used in this study come from the Kenneth kraemer's lab (Laboratory of Cancer Biology and Genetics, Center for Cancer Research, National Cancer Institute, Building 37 Room 4002 MSC 4258, Bethesda, MD 20892 -4258) and from the Donata Orioli's lab (IGM-CNR, Via Abbiategrasso, 207 - 27100 Pavia, Italy).
These cell lines have been previoulsy described (see refs in the paper):
382BE (normal fibroblasts isolated from the mother of TTD379BE) (ref 25)
TTD379BE (bearing the TFIIEβ/A150P point mutation) (ref 25)
C3PV (control cell line for TTD28PV) (ref 29)
TTD28PV (bearing the TFIIEβ/D187Y point mutation) (ref 29)
TTD8PV father (control cell line for TTD8PV) (ref 57)
TTD8PV (bearing the XPD/R112H point mutation) (ref 57)
TTD12PV father (control cell line for TTD12PV) (ref 58)
TTD12PV (bearing the XPD/R722W point mutation) (ref 58)
In parallel, U2OS IIEβ/WT (human osteosarcoma cell line) have been used (ATCC HTB-96) to generate U2OS IIEβ/A150P. Clones were screened by PCR and enzymatic digestion followed by further sequencing to confirm the correct knock-in in the desired locus (as described in the manuscript, Material and Methods, see also Supplementary Fig.2)

Authentication	see above for more information
Mycoplasma contamination	all cell lines tested negative for mycoplasma contamination.
Commonly misidentified lines (See ICLAC register)	No cell lines used are listed in the database of commonly misidentified cell lines.
